# Recovery-Oriented Intersectoral Care in Mental Health: As Perceived by Healthcare Professionals and Users

**DOI:** 10.3390/ijerph17238777

**Published:** 2020-11-26

**Authors:** Kim Jørgensen, Tonie Rasmussen, Morten Hansen, Kate Andreasson, Bengt Karlsson

**Affiliations:** 1 The Research Collaboration, Psychiatric Centre North Zealand, Dyrehavevej 48, DK-3400 Hilleroed, Denmark; kate.aamund@regionh.dk; 2 Center for Quality and Development, Department of Social Health, Rudersdal Kommune, Stationsvej 36, 3460 Birkerød, Denmark; tora@rudersdal.dk; 3 Psychiatric Outpatient Clinic Nørrebro Griffenfeldsgade 46, 2200 Copenhagen, Denmark; aesel82@gmail.com; 4 Center for Mental Health and Substance Abuse, Department of Health, Social and Welfare Studies, Faculty of Health and Social Sciences, University of South-Eastern Norway, Postbox 7053, 3007 Drammen, Norway; bengt.karlsson@usn.no

**Keywords:** nursing care, healthcare professionals, users, recovery-oriented, intersectoral care, mental health hospitals, community mental healthcare, collaboration, patient participation

## Abstract

This study aimed to explore how mental health professionals and users perceive recovery-oriented intersectoral care when comparing mental health hospitals and community mental healthcare. Methodological design: Five audio-recorded focus group interviews of nurses, other health professionals and users were explored using manifest and latent content analysis. Ethical issues and approval: The study was designed in accordance with the ethical principles of the Helsinki Declaration and Danish law. Each study participant in the two intersectoral sectors gave their informed consent after verbal and written information was provided. Findings: From the health professionals’ perspective, the main theme informed by subthemes and categories was formulated: ‘Recovery-oriented intersectoral care requires more coordination and desire for collaboration’. Two subthemes were subsequently formulated: ‘The users´ perspective of the centre’ and ‘Need for a common agenda and understanding of recovery-oriented intersectoral care’. From the users´ perspective, the main theme was formulated as: ‘Recovery-oriented intersectoral care in tension between medical- and holistically oriented care’. This theme was informed by two subthemes: ‘The users´ perspective is not in focus’ and ‘A trusting relationship and a holistic approach brings coherence’. Conclusions: This study reveals that health professionals want to work in a recovery-oriented manner in intersectoral care, but several challenges appear which make achieving this aim difficult. A common understanding of recovery and how it should be carried out in intersectoral care does not exist. Care decisions are primarily made paternalistically, where the users’ and relatives’ voices are ignored. In an attempt to create coherence across sectors, intersectoral network meetings have been established with health professionals from both sectors. However, the meetings are characterised by a lack of a clear purpose regarding the meeting structure and content, and users are only minimally involved. Our results can contribute to dealing with the challenges of incorporating recovery-oriented intersectoral care as an ideology in all psychiatric and municipal contexts and is, therefore, important for health professionals and users.

## 1. Introduction

Recovery-oriented intersectoral care in mental health is a cornerstone in the debate concerning government health policy in Scandinavia and other Western countries. Recovery can be considered as being in conflict with a paternalistic ideology, where the patient is considered a passive recipient of care [1]. Today, the focus must be on putting the user at the centre of care. Specifically, this involves giving him or her the opportunity to gain the strength and independence to be independent of professional help and to have attained self-support [2].

Research shows different definitions of recovery, defined for example, as ‘a deeply personal, unique process of changing one’s attitudes, values, feelings, goals, skills, and/or roles’. It is a way of living a satisfying, hopeful, and contributory life even within limitations caused by illness. Recovery involves ‘the development of new meaning and purpose in one’s life as one grows beyond the catastrophic effects of mental illness’ [3]. Personal recovery has emerged from the consumer movement in the past 20 years, in response to paternalistic and medically oriented psychiatry [4,5]. Personal recovery is often referred to in policy documents [6], but, in clinical practice, clinical recovery is often seen as the dominant approach to care and treatment [7,8]. Clinical recovery generally refers to clinical outcomes where the focus is on the symptoms of illness and the absence of disease, or on a cure [9]. Recovery and intersectoral care are often linked to policy documents and government goals, where both recovery and intersectoral care are mentioned as approaches on which mental health should be based [10,11]. According to the English IMROC [The Implementing Recovery through Organisational Change] environment, the creation of a recovery-oriented culture in mental healthcare presupposes an overarching strategy, supported by training and awareness-raising for staff, people using the services, and those close to them. Within this context, the Team Recovery Implementation Plan becomes a means for individual teams to make recovery a reality on the frontline [12]. However, there is limited research exploring the relationships between recovery and intersectoral care. In addition, there is a lack of research showing professionals’ and users’ experiences of intersectoral care based on a recovery-oriented framework [13,14]. Much of the research related to intersectoral care focuses only on the transition itself, e.g., discharge from the mental health hospital to community mental healthcare [15,16,17]. In addition, there are several studies exploring methods for creating better intersectoral care. The results of this part of the research show that the sector is mainly focused on the short-term recovery of the patient and not on long-term intersectoral care by the mental health hospital and community mental healthcare [18,19,20]. Some studies show that the mental health hospital and community mental healthcare are not based on a holistic approach to treatment and rehabilitation. The professionals in both sectors are convinced that they work in a recovery-oriented way and have a holistic perspective on care [2,21]. Research shows that the professionals in mental health hospitals primarily focus on diagnosis, symptoms, medicine, and self-care.

As a consequence, the users’ perspective can be overlooked. The treatment and rehabilitation in other forms of intersectoral care are subject to professional steering and constrain the involvement of the users’ wishes [6,22,23]. Moreover, research that shows how the professionals should work in a recovery-oriented manner in intersectoral care is often vague [24]. The lack of clarity from the healthcare professionals, and user perceptions about recovery-oriented intersectoral care in mental health, mean that healthcare professionals focus on users’ difficulties in the here and now and do not relate these to long-term care where the users’ perspectives are involved. This study aims to explore how mental health professionals and users perceive recovery-oriented intersectoral care carried out by mental health hospitals and community mental healthcare in Denmark.

## 2. Methods

The study’s epistemology focuses on mental health professionals’ and users’ perceptions of recovery-oriented intersectoral care carried out by mental health hospitals and community mental healthcare. A qualitative design was used to gather in-depth knowledge about participants’ perceptions. We used qualitative content analysis to generate themes from five focus group interviews [FGIs] [25]. In the content analysis, the audio-recorded FGIs were transcribed verbatim and carefully read through several times [26,27]. We identified patterns in the data by dividing them into meaning units, and we created groups of expressed manifest content with shared commonality.

### 2.1. Recruitment and Sampling

An invitation was sent to the management of the mental health hospital and of community mental healthcare. A purposive sampling technique [28] was used to establish contacts and recruit participants for this study. The management provided contacts, and then the first author contacted the healthcare professionals, who assisted in gathering participants for the FGIs.

The health professionals helped to communicate with users and examined whether some would participate in a FGI. In community mental healthcare, there was a network for users who wanted to participate. In the mental health hospital, there was a group of users who would soon be discharged and were interested in sharing their experiences.

All the participants had to have experience and/or knowledge of the intersectoral collaboration between psychiatric centres and community mental healthcare in order to be eligible for the study. Users’ eligibility rested on them having experience with admissions in both psychiatric centres and community mental healthcare. In total, 27 informants agreed to participate, nine of whom represented a user perspective. Among the healthcare professionals, 14 assisted with treatment in outpatient or in-patient psychiatric centres and four were employed in community mental healthcare [Table 1].

### 2.2. Data Collection–Focus Group Interviews

Data were collected through FGIs to obtain different views on the topic. A collective vocabulary can elicit more spontaneous, expressive and emotional views than when using individual interviews [27]. In addition, group interaction can make it easier to express opinions that are usually withheld when subjects are confronted with sensitive, taboo topics [27,29]. To gain a deeper insight from the FGIs, we wanted to bring together 5–8 participants in each FGI. We managed to gather between 4–7 participants in each focus group [Table 1]. No participants dropped out.

The FGIs were conducted with a set time of one hour and were conducted and facilitated by the first, fourth and fifth authors, in the environment preferred by the participants. While one was in charge of creating a dynamic dialogue and being a moderator, the others provided assistance in asking in-depth questions and validating the content at the end of the discussions [30].

We used a topic guide [Table 2] as an aid to address important topics and thus gain rigorous knowledge about the participants’ perceptions of intersectoral care [27,31].

The FGIs were audiotaped and lasted approximately 60 min in duration, were transcribed verbatim by a research assistant, and amounted to 70 pages. All names in the transcripts presented here have been anonymised.

In the research group, we reflected on and discussed our positions in the research field, as well as our perceptions. All authors had widespread experience in the psychiatric field as nurses, one physician, one educator, and one with user experience. Three authors have PhDs, and all authors were active in different projects relating to mental healthcare.

### 2.3. Data Analysis

In this study’s epistemology, we developed themes from a qualitative content analysis of FGIs. The content analysis contains two levels. First, an analysis of the manifest content was performed. Here, the focus is on what the participants in the FGIs perceive, and only a small degree of interpretation is used. The second level concerns the latent content in which data are processed and interpreted. The analysis of the latent content seeks an understanding of the meaning of the elements of the informants’ narratives [25]. The content analysis consists of four stages: condensed meaning unit, category, sub-theme and theme. The condensed meaning units were abstracted and labelled with a code. Long statements were summarised into shorter statements where the main meaning of what had been said was reworded as succinctly as possible. The different codes were compared and sorted into categories, i.e., a group of content that shares commonality and constitutes the manifest content. One category answers the question ‘What?’ [32] and can be identified as a thread through the codes. The preliminary categories were discussed in the research team and were subdivided into multiple meanings, as performed in several subthemes and themes at different levels of abstraction [Table 3].

### 2.4. Ethical Considerations

This study was conducted in adherence to scientific ethics. The Danish Knowledge Centre approved the study for Data Reviews [J.nr.: P-2019-753]. According to the Helsinki Declaration [33] and Danish law [34], no formal permit from a biomedical ethics committee was required, as the purpose of the research was not to influence the informants, either physically or psychologically.

Informed consent was obtained from all participants after they had received verbal and written information about the purpose of the study. The participants were informed that their participation could be halted at any time and that all data would be treated so that no unauthorised person would have access to the material. Data were treated confidentially and anonymously, and voice files were erased after transcription of the material. All other data material will be destroyed after publication. The users in this project can be considered to be vulnerable. Therefore, there are extended ethical commitments in ensuring an equivalent, transparent and participatory research collaboration [35]. Consequently, we met the users one hour before the FGI to have a dialogue regarding the project and give them plenty of time to consider their participation. Two users chose to follow up with their community healthcare professionals through the FGI, which the other participants agreed to.

## 3. Results

First, we will present the results from the health professionals’ perspective and, hereafter, from the users’. In reflecting how mental health professionals perceive recovery-oriented intersectoral care between mental health hospitals and community mental healthcare, the main theme informed by subthemes and categories was formulated: ‘Recovery-oriented intersectoral care requires more coordination and collaboration’. Two subthemes were subsequently formulated: ‘The users´ perspective on the centre’ and ‘Need for a common agenda and understanding of recovery-oriented intersectoral care’, which, along with the categories, will subsequently be unfolded. Direct quotations from the FGIs were selected to illustrate the development of the most commonly reported aspects of each category and subtheme.

### 3.1. The Users’ Perspective on the Centre Perceived by Health Professionals

#### Focus on Collaboration and User Needs

Eighteen participants from both sectors think that recovery-oriented intersectoral care succeeded when they focused on it. This is very much about giving priority to collaboration across sectors and putting users’ recovery process at the centre. In addition to relevant health professionals meeting with users, there is also a need to bring together all health professionals across sectors and focus on recovery and collaboration. One participant stated:

*‘It creates coherence when mental health hospital and community mental healthcare meet every quarter for three hours with all professionals relevant to the user’s intersectoral care’ [3].*

Half of the participants from the mental health hospital perceive that the possibilities of intersectoral care also depend on the attitude to cooperation shown by the individual health professionals.

*‘We are dependent on which health professionals from the municipalities come to the meeting about what support the user is offered’ [1].*

Willingness to meet colleagues and users is thus also conditional on the specific nurse or educator’s attitude, and, in addition, an economic aspect was evident. In Copenhagen, there are 29 municipalities, some of which are wealthy. In contrast, others are more socially disadvantaged, which is reflected in the resources each municipality allocated to collaborative meetings and conducting recovery-oriented support, etc.

*‘Some Municipalities Have a Better Economy and Offer More Opportunities for Help and Activities for Users’ [1].*

#### Focus on Recovery-Oriented Intersectoral Care and Rehabilitation

Fifteen participants perceive recovery as something that users themselves must do; however, healthcare professionals must support the users’ recovery process and it is a collaboration where they show that they believe that an individual can recover from a mental illness.

*‘We may have a rehabilitative approach or attitude. It is not for the users but with the users. It is essential for us’ [2].*

Recovery-oriented work involves putting the user’s perspective at the centre and examining how intersectoral care can be targeted at users’ needs for help.

*‘[….] To be able to support them at all, we have to ask what they have in mind and their wishes for the future’ [2].*

The majority of participants had experienced a need to help users build social communities where they felt included. Users can easily be stigmatised as merely a diagnosis, but they need to feel like a normal human being with identities such as that of boyfriend, parent, football player, student, etc.

*‘Having a work identity really matters whether you are a volunteer, get a salary, whether it is a flexible job or whatever it is, it has a really big impact on what it feels like to feel that you can do something for someone or something’ [2].*

### 3.2. Need for a Common Agenda and Understanding of Recovery-Oriented Intersectoral Care

#### Different Expectations for Cross-Sectoral Meetings

Sixteen participants agreed that intersectoral network meetings about users’ care are important and must be prioritised. However, the participants do not agree on the aims and content of the meetings. Participants from both sectors report that the meetings are often about users’ medicine and search for housing, disability pension, etc. The users’ voices and wishes, their hopes and aims are not discussed but are implicitly included in the help measures the system must offer.

*‘I have also been called to such a network meeting where we just have to step up, and we simply sit around such a group, and there is a single user, and it is in the middle of a course, and then it is all about medicine’ [2].*

Network meetings open up the opportunity to share knowledge about users and what help can be offered across sectors.

*‘I think there have been some really good meetings that we have been to once in a while where they have needed our knowledge of the users, how they were habitually’ [2].*

#### Long-Term Intersectoral Coordination Is Deficient

Seventeen participants from both sectors believe they lack continuity across handovers. The plans made at the hospital and the municipality rarely go any further, and users start all over again every time.

*‘But when they walk out the door here, the interim plan ends very abruptly. It is not so that we pass it on to a municipality and say: We have worked for this. And who should we give it to? I think that sometimes is our challenge’ [1].*

One of the problems is that the sectors do not share a communication system, so the user’s history and plans are not passed on from one sector to another.

*‘Yes, if you had a system where everyone could go in and read: what have we been working on with the user? For example, we also make user plans in nursing. And it could be very relevant that maybe there was a housing aid that could read it later, and then continue with the goals here that we have had and the actions and so on. I think there is a lack of somewhere to gather data around which can be continued in the primary sector’ [1].*

*‘In the hospital sector, you work in an interdisciplinary way and try to have a structure. But when you move into the municipal image, the job centre is one size. Those who grant housing offer a different size. If you also need an aid, then this is the third size. And it is almost impossible to move in it’ [3].*

#### Focus on Health Professionals’ or User Needs

Eight participants pointed out a problem of many questions and decisions being made from the perspective of professionals. In the mental healthcare hospital, the primary focuses are diagnoses and medicine. In community mental healthcare, the focuses are on achieving self-care, self-sufficiency, own apartment, etc.

*‘We also get many of the patients who have no insight into the disease and who do not think they need any help, but where we think it is needed’ [1].*

*‘[….] In the municipality, there are paragraphs… 82 is a temporary support for half a year, and 85 is a little longer-term support, then the municipality makes goals with the user, so he becomes responsible for his semi-annual social pedagogical efforts, can more easily take ownership of what needs to happen. So, it will not be us and our goals–it will be the user’s own goals’ [2].*

It appears that the participants have many ideas on what problems the users have and how to handle these problems. The user’s perspective seems to step into the background.

*‘There is an incredible number of people among some of our patients who have been here for many years. And we all have an idea of what’s going to happen. Both in the ward and in the outpatient care, they may think otherwise. In the emergency room, they may have seemed third when he or she entered. And then the municipalities may seem quite fourth’ [1].*

### 3.3. The Users’ Perspective Is Not in Focus-Perception of the Users

#### Clashes between Mental Health Hospitals and Community Mental Healthcare and the User as a Hostage

In reflecting the participants’ perceptions of recovery-oriented intersectoral care, the main theme was formulated as ‘Recovery-oriented intersectoral care in the tension between medical- and holistic-oriented care’. This theme was informed by two subthemes: ‘The users’ perspective is not in focus’ and ‘A trusting relationship and holistic approach brings coherence’, and these categories will be subsequently unfolded [Table 4].

Different health professionals cross the two sectors, e.g., psychologists, physicians, therapists, and nurses do not agree on the organisation of future plans.

*‘My psychologist and doctor said I should not be in the job market right now. A job consultant in the municipality said it was good for me to work, and my doctor did not understand the labour market’ [4].*

Another problem is that the health professionals do not talk together about the user recovery process, and the user becomes a hostage to the professionals’ lack of communication.

‘I have many different therapists in the job centre, resource course or other and the communication becomes difficult, and they do not get talking together about my course’ [4].

Users are asking for help and involvement and are being offered medicine instead

The participants were particularly critical of previous admissions to mental health hospitals, and, despite offering psychosocial coping strategies, it was mainly medication that was offered to solve problems in the here and now.

*‘You get a safety plan when you are hospitalised. There I have written a lot of strategies that I can do for my voices and anxiety and paranoia and all that. But they just stuff me with medicine instead’ [5].*

The participants do not experience a focus on their recovery process across sectors, and the medical treatment paradigm undermines their own perspectives on life.

*I have some opinions about the world and life, and, when I tell the staff about it, they say: You just need some more pills [4].*

*The doctor filled me with medication, and I slept all the time. I said to him: Tell me, do you want me to sleep my life away? Yes, the doctor said [5].*

### 3.4. A Trusting Relationship and Holistic Approach bring Coherence

Municipal professional support as a fixed point

The participants perceive that flexibility is essential, so they obtain the necessary help after discharge from the mental healthcare hospital, when many things have to work again in everyday life.

*‘My personal healthcare professional in community mental healthcare helps when I need it if there is something’ [4].*

Good relationships with healthcare professionals are essential to maintaining hope and belief in recovery and to lead a good life.

*‘I have my personal healthcare professional in community mental healthcare for many years, if I did not have her, I would not be alive today’ [4].*

Six participants had positive experiences of being associated with a through-person health professional in the municipality who, among other things, could also be a companion to meetings, e.g., if the user were admitted.

*‘When I have been admitted, my personal healthcare professional in community mental healthcare has always been there. And she also came and visited me in the hospital. It’s been really nice’ [5].*

*‘If I had not had my personal healthcare professional in community mental healthcare, I would have been left to myself as I was at the beginning before I got my housing allowance’ [5].*

### 3.5. Relative Involvement

Involving relatives in intersectoral care can partially help to bring important stories forward and keep the focus on recovery. Moreover, the involvement of relatives promotes their understanding of what it means to live with a mental illness. This insight enables the relatives to be better at understanding and helping the person with mental difficulties.

*‘My sister has been involved. It’s been really good. Then they got an understanding of what was really wrong because I found it difficult to put words to it myself’ [5].*

*‘I want them to be able to understand the things I say about it. Not the disease itself but understand when I explain’ [5].*

## 4. Discussion

This study aimed to explore how mental health professionals and users perceived recovery-oriented intersectoral care between mental health hospitals and community mental healthcare. The health professionals perceived that recovery-oriented intersectoral care required more coordination and collaboration. From a user perspective, recovery-oriented intersectoral care was perceived as the tension between medical- and holistic-oriented care.

First, we discuss two themes oriented from the health professionals’ perspective: ‘The users’ perspective of the centre’ and ‘Need for a common agenda and understanding of recovery-oriented intersectoral care’. Hereafter two themes from the users´ perspective are discussed: ‘The users’ perspective is not in focus’ and ‘A trusting relationship and holistic approach brings coherence’. These four themes will form the subheadings in the following discussion.

### 4.1. Mental Health Professionals’ Perceptions of Recovery-Oriented Intersectoral Care between Mental Health Hospitals and Community Mental Healthcare

The results reflect the professionals’ self-criticism and the barriers to creating good recovery-oriented intersectoral care. As a result, there is a need for better cross-sectoral coordination and for all health professionals’ to show a willingness to collaborate, focus on users’ recovery and involve them in decisions about care, treatment and rehabilitation.

### 4.2. The Users’ Perspectiveoft the Centre

Sixteen participants think the prerequisite for successful recovery-oriented intersectoral care was to prioritise all relevant health professionals participating in meetings with users. Inadequate time appears the cause of the intersectoral meetings not working. These meetings should focus on the user’s hopes and wishes for the future and how health professionals can promote the user’s recovery process. Another problem was that mental health professionals reported that municipal contact persons from the nine cooperating municipalities did not always participate, despite everyone having good intentions and the wish to create coherence and ensure a better future after discharge.

Participants reported that it depended a great deal on what attitude the individual health professionals showed, whether it was to the user’s recovery process or to the professionals’ goal of, e.g., full symptom remission, full- or part-time work or education, or independent living without supervision by informal carers. Leading researchers in recovery also speak in favour of centring the professional help on the users’ perspective and introducing recognised psychosocial approaches to recovery, under terms such as personal recovery, recovery in, or recovery with a large R [5,36,37]. Our results show that participants from both sectors think intersectoral meetings must focus on the user’s hopes and wishes for the future and how health professionals can promote the user’s recovery process. All participants perceived recovery-oriented intersectoral care as important and the right approach to care, but, in clinical practice, many barriers appear to prevent it. As in other studies [21], barriers serving to hinder a good intersectoral collaboration included restricted finances and too few staff resources to achieve an effective intersectoral collaboration with a focus on recovery. It is not sufficient to create and maintain intersectoral meetings; there is a need for a common understanding of what these intersectoral meetings should address and what recovery-oriented care means in a clinical practice setting.

### 4.3. Need for a Common Agenda and Understanding of Recovery-Oriented Intersectoral Care

The results show that the achievement of recovery-oriented intersectoral care requires health professionals to agree on a common purpose and agenda for the intersectoral meetings. In these meetings, the following professionals take part: nurses, doctors, social workers and educators. These meetings are called network meetings because the users and relatives are invited. It is far from all relatives who attend the meetings, but the users often attend. The network meetings are often held during user hospitalisation and revolve around treatment in the here and now. There is no focus on long-term intersectoral care and recovery is oriented towards symptoms and achieving self-care and self-sufficiency. Previous studies confirm that care will be concerned with achieving the professionals’ goal of making the user independent of professional help, and there is a lack of focus on users’ personal recovery [8]. Western psychiatry is governed by neoliberal thinking where the focus is on making users themselves responsible for their recovery and able to cope with their problems [2,38]. The health system is individualised; thus, the user is made into a consumer who, for example, must attend networking meetings and articulate their need for help to recover from their disorder.

Another problem is that, despite health professionals’ willingness to work in an intersectoral manner, they are predominantly preoccupied with solving the problems they encounter with users in the here and now. The consequence is that mental health professionals mainly focus on the medical treatment of symptoms. In contrast, community mental healthcare focuses on whether the user can profit from some of the legal benefits that exist. Researchers have previously called this a silo structure approach that reflects a barrier to focusing on intersectoral long-term care [14,39,40]. There are many indications that recovery-oriented intersectoral care requires health professionals from both sectors and users and relatives in the community to focus on how the user wishes the recovery process to be supported professionally [11,41]. The results show that the plans prepared in one sector do not follow in the next sector and that the process is started from scratch every time. In some Western countries, new ways of organising psychiatry are being introduced with the goal of promoting a recovery-oriented culture. User-controlled beds, hiring peers and more follow-up help after discharge are among the recent initiatives to promote a recovery-oriented and user-involved culture.

### 4.4. Users’ Perceptions of Recovery-Oriented Intersectoral Care between Mental Health Hospitals and Community Mental Health Care

As a result, recovery-oriented intersectoral care occurs in a field of medical- and holistically oriented care. Users predominantly experience that the medical approach prevails, which is mainly addressed in mental health hospitals.

### 4.5. The Users’ Perspective Is Not in Focus

The users become hostages in a system where health professionals do not agree on a plan for the recovery process. Users’ own perspectives are overlooked, and their fate is subject to the professionals’ paternalistic decisions. Users lack involvement in their course and feel that their perception of problems and wishes for recovery is not listened to. Users are subject to clinical recovery, which is ill an approach, which other studies demonstrate, dominating Western mental healthcare [38,41]. Several studies explain the maintenance of a paternalistic control as part of the development of society, where, for example, economy, efficiency and medical evidence are more important as orientation than the user’s own experiences and opinions [13]. Another problem is that users are retained under medical treatment despite submitting psychosocial plans for how they can cope with various mental challenges. Medical treatment becomes the first choice to solve the users’ mental difficulties, which is also a trend seen in other recent studies. The problem is that both mental health hospitals and community mental healthcare must work in a recovery-oriented manner [2]. However, there is no agreement on what recovery means and how it should be performed in clinical practice. The consequence is that users are exposed to what the individual nurse or doctor thinks is important, which is not necessarily recovery-oriented.

### 4.6. A Trusting Relationship and Holistic Approach bring Coherence

In community mental healthcare, many users with severe mental difficulties are given a contact person as professional support, usually an educator, as someone who follows them for a part of the time in the user’s everyday life. It means a great deal to the users to have a relationship with a professional who follows them and who is helpful in providing both psychological support and help with practical issues. An important aspect is that these health professionals offer flexible help when it fits into the user’s everyday life. This also helps to create coherence between the sectors because the contact person follows the user if, for example, he or she is admitted. Some users also see this contact person as a friend with whom they share many stories and who helps prevent loneliness. This result supplements previous studies, which reflect problems with many changing contacts in users’ intersectoral care. Frequent changes of professionals have consequences for the relationship, and many stories are lost and have to be retold again and again, which damages the continuity of the users’ care [1].

Users demand more involvement of relatives, as this would provide them with knowledge about mental disorders and thus increase their understanding. Furthermore, the relatives would be an important point of support, and they can help create coherence. As researchers point out, the involvement of relatives must not only remain in the terms of the system. Relatives’ involvement often turns out to focus on relieving the professionals and supporting the neoliberal idea of enabling users to take responsibility for their own health, rather than a collective responsibility to provide the user with maximum psychosocial recovery-oriented support [11]. Some users do not want the involvement of their relatives, which must be respected, and some do not have relatives or no longer have contact with them, with implications for practice.

The results indicate that health professionals from both sectors must prioritise a close dialogue between the sectors about users’ intersectoral care. Network meetings can form the basis for a structure via which the relevant professionals meet with the user and relatives. There is a need for a clarification of how recovery-oriented intersectoral care is understood and should be performed and what the purpose should be in the network meetings. The results show users’ need for more involvement of both themselves and their relatives in shared decision-making.

### 4.7. Methodological Considerations

To ensure the validity of this study, we carefully selected rigorous data material and systematically treated it to an in-depth exploration using manifest and latent content analysis. The empirical material was rigorous as we achieved a wide variety of both health professionals and users recruited from both sectors. The results are in line with the existing research. They are transferable to clinical practice as they contribute to a greater understanding of the challenges associated with healthcare professionals’ and users’ perceptions of recovery-oriented intersectoral care between mental health hospitals and community mental health care.

## 5. Conclusions

Health professionals from mental health hospitals and community mental health care always wish users the best in terms of recovery. However, they have no common understanding of recovery or tools. This study reveals that health professionals want to work in a recovery-oriented manner in intersectoral care, but several challenges appear when trying to achieve this aim. No common understanding exists of recovery and how it should be carried out in intersectoral care, and decisions about care are primarily made on a paternalistic basis, where the users’ and relatives’ voices are ignored. In an attempt to create coherence across the sectors, intersectoral network meetings have been established with health professionals from both sectors. However, the meetings are characterised by a lack of a clear purpose in the meeting structure and content, and users are minimally involved.

## Figures and Tables

**Table 1:** Overview of the focus groups.

Focus Group Number	Context	Number of Participants Per Focus Group
1	Mental health hospital	Healthcare professionals *n* = 7
2	Mental health hospital	Healthcare professionals *n* = 7
3	Community mental healthcare	Healthcare professionals *n* = 4
4	Mental health hospital	Users *n* = 4
5	Community mental healthcare	Users *n* = 5
	TOTAL NUMBER OF PARTICIPANTS: 27	

**Table 2:** Taboo Topics.

General Perceptions and Experiences of Intersectoral Care in Mental Health	Examples of Intersectoral Care
	User participation
	Trust
	Communication
	Relatives
	Continuity and coherence
Short resumé	Examples of experiences in context
	Structure
	Continuity
	Transitions
	Transitions
	Individually focused
	Meanings/confidences/relationships
Final summary	

**Table 3:** Condensed meaning units, categories, subthemes, and themes perceived by health professionals.

Condensed Meaning Unit	Category	Subtheme	Main Theme
It created coherence when the mental health hospital and community mental healthcare meet every quarter for three hours with all professionals relevant to the users’ intersectoral care [3]. Some municipalities have a better economy and offer more opportunities for help and activities for users [1]. We are dependent on which health professionals from the municipality come to the meeting regarding what support the users are offered [1].	Focus on collaboration and users’ needs	The users’ perspective at the centre	Recovery-oriented intersectoral care requires more coordination and desire for collaboration
We don’t work with recovery because you can’t work with that. Recovery is something users themselves must have or create [2]. We may have a rehabilitative approach or attitude. It is not for the users but with the users. It is essential for us [2]. The users must be at the centre. And in order for us to be able to support them at all, we have to ask what they have in mind and their wishes for the future [2]. Having a work identity really matters whether you are a volunteer, have a salary, whether it is a flexible job or whatever it is, it has a really big impact on what it feels like to feel that you can do something for someone or something [2].	Focus on recovery-oriented care and rehabilitation		Recovery-oriented intersectoral care requires more coordination and desire for collaboration Recovery-oriented intersectoral care requires more coordination and desire for collaboration
I have also been called to such a network meeting where we just have to turn up and we simply sit around such a group and there is a single user, and it is in the middle of a course, and then it is all about medicine [2]. I think there have been some really good meetings that we have been to once in a while where they have needed our knowledge of the users, and how they were habitually [2].	Different expectations for cross-sectoral meetings	Need for a common agenda and understanding of recovery-oriented intersectoral care Need for a common agenda and understanding of recovery-oriented intersectoral care	
But when they walk out the door here, the interim plan ends very abruptly. It is not the case that we pass it on to a municipality and say: We have worked for this. And who should we give it to? I think that sometimes is our challenge [1]. Yes, if you had a system where everyone could go in and read: What have we been working on with the user? For example, we also make user plans in nursing. And it could be very relevant that maybe there was a housing aid that could read it later, and then continue with the goals here that we have had and the actions and so on. I think there is a lack of somewhere to gather data around, which can be continued in the primary sector [1]. In the hospital sector, you work in an interdisciplinary way and try to have a structure. But when you move into the municipal image, the job centre is one size. Those who grant housing offer a different size. If you also need an aid, then this is a third size. And it is almost impossible to move in it [3].	Long-term intersectoral coordination deficiency		
We also get many patients who have no insight into the disease and who do not think they need any help, but where do we think it is needed? [1]. In the new year, there will be two paragraphs for us to use. There is an 82 and an 85. 82 is temporary support for half a year; 85 is slightly longer-term support. For 82 support, you scan up from the administration together with the user - ‘how do you fit in here?’. But there are no goals. Then we start with an open-dialogue meeting, where we talk to the user: On what issues do you want to work with us for the next six months? So, the user himself becomes responsible for his semi-annual social pedagogical efforts, to make it easier also for the user himself to take ownership of what is to happen. So, it will not be us and our goals–it will be the user’s own goals [2]. We can say that the user is poor and has low-level functioning, is poor at managing personal hygiene and finds it difficult to act and has no network, and then the case manager should, like, say: It sounds like you need a professional’s support. Just as if they didn’t say: Why don’t you give that user some assistance which is more than medicine? A doctor here would also think it was quite provocative [1]. There are an incredible number of people among our patients who have been here for many years. And we all have an idea of what’s going to happen. Both in the ward and in the outpatient care, they may think otherwise. In the emergency room, they may have seemed to have been the third need when he or she entered. And then, the municipalities may seem quite fourth [1].	Focus on health professionals’ or users’ needs		

**Table 4:** Condensed meaning units, categories, subthemes, and themes as perceived by users.

Condensed Meaning Unit	Category	Subtheme	Main Theme
My psychologist and doctor say I should not be in the job market right now. A job consultant in the municipality said it was good for me to work. My doctor did not understand the labour market. I have many different therapists in the job centre, at the resource course, or in other facilities. The communication becomes difficult, and they do not get to talk together about my course [4].	Clashes between mental health hospitals and community mental healthcare hold the user hostage	The users’ perspective is not in focus	Recovery-oriented intersectoral care in tension between medical- and holistically oriented care Recovery-oriented intersectoral care in tension between medical- and holistically oriented care
You get a crisis plan when you are hospitalised. There I have written a lot about strategies that I can do for my voices and anxiety and paranoia and all that. But they just stuff me with medicine instead [5]. I have some opinions about the world and life, and when I tell the staff about them, they say: You just need some more pills [4]. The doctor filled me with medication, and I slept all the time. I said to him: Tell me, do you want me to sleep my life away? Yes, the doctor said [5].	Users are asking for help and involvement and are being offered medicine instead		
My personal healthcare professional in community mental healthcare helps when I need it if there is something wrong [4] I have had my personal healthcare professional in community mental healthcare for many years. If I did not have them there, I would not be alive today [4]. When I have been admitted, my personal healthcare professional in community mental healthcare has always been there. And she also came and visited me in the hospital. It’s been really nice [5]. If I had not had my personal healthcare professional in community mental healthcare, I would have been left to myself as I was at the beginning before I got my housing allowance [5].	Municipal professional support as a fixed point	A trusting relationship and holistic approach bring coherence	
My sister has been involved. It’s been really good. Then they got an understanding of what was really wrong because I found it difficult to put words to it myself [5]. I want them to be able to understand the things I say about it. Not the disease itself, but to understand when I explain [5].	Relative Involvement		
